# Ghost Cells as a Two‐Phase Blood Analog Fluid—Optical Thrombus Growth Detection Using Particle Image Velocimetry

**DOI:** 10.1111/aor.15042

**Published:** 2025-06-18

**Authors:** Benjamin J. Schürmann, Pia Creutz, Thomas Schmitz‐Rode, Ulrich Steinseifer, Johanna C. Clauser

**Affiliations:** ^1^ Department of Cardiovascular Engineering, Institute of Applied Medical Engineering University Hospital RWTH Aachen University Aachen Germany; ^2^ Institute of Applied Medical Engineering University Hospital RWTH Aachen University Aachen Germany

**Keywords:** particle image velocimetry, resealed ghost cells, thrombus growth monitoring, translucent two‐phase blood analog fluid

## Abstract

**Background:**

In vitro thrombosis tests for mechanical circulatory support systems lack standardized ISO guidelines. A major limitation of current approaches is the absence of continuous thrombus monitoring, as terminated experiments at a single time point fail to capture the dynamic nature of thrombus formation. However, spatially resolved thrombus formation and its underlying dynamics are crucial for the optimization of mechanical circulatory support systems.

**Methods:**

In this study, we present a high‐resolution thrombus monitoring approach using particle image velocimetry with a thrombogenic, two‐phase blood analog fluid, designated as “ghost blood”. Ghost blood consists of plasma and ghost cells, which are hemoglobin‐depleted erythrocytes. We validate and quantify the particle image velocimetry with ghost blood and use this combination to monitor thrombus growth.

**Results:**

The validation demonstrated velocity fields in the FDA‐pump are consistent with existing literature, confirming the usability of ghost blood in particle image velocimetry. The use range of ghost blood is quantified as a formula to determine the maximum possible optical penetration depth. Finally, thrombus growth was successfully monitored in the FDA‐pump.

**Conclusion:**

In this proof of principle study, we grew a thrombus in the FDA‐pump and were able to monitor its growth from a first thrombus thread to a complete obstruction of the flow. This approach enables both the localization and the temporal growth of the thrombus to be visualized and thereby provides a foundation for future advancements in thrombosis assessment and the optimization of mechanical circulatory support systems.

## Graphical abstract

Ghost cells as two‐phase blood analog fluid enable us to monitor thrombus growth through the implementation of particle image velocimetry. In this proof of principle study, we grew thrombi in the Food and Drug Administration pump and were able to monitor their growth from a first thrombus thread to a complete obstruction of the flow.

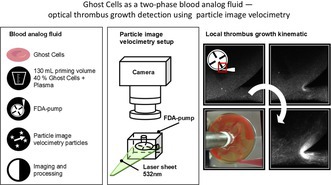

**Abbreviations**
ACT: Activated clotting time
FDA: Food and Drug Administration
GB: Ghost blood, ghost cells combined with plasma
GCs: Ghost cells
MCS: Mechanical circulatory support systems
OPD: Optical penetration depth
OPD_Camera_
: from the seeding particles to the camera
OPD_Laser_
: from the laser to the seeding particles
PBS: Phosphate‐buffered saline
PIV: Particle image velocimetry
SNR: Signal‐to‐noise ratio

## 1 Background

Mechanical circulatory support systems (MCSs) face two major challenges: hemolysis and thrombosis. Currently, in vitro thrombosis tests for MCS lack standardization in ISO guidelines [1]. While multiple groups are working on thrombosis tests for MCS [2, 3, 4, 5, 6, 7], their approaches involve circulating blood through the MCS until an experiment termination condition is met. These termination conditions vary between the visual detection of thrombus formation or significant changes in systemic parameters (e.g., pressure) or blood parameters (e.g., clotting time). Upon experiment termination, the MCS is flushed with phosphate‐buffered saline (PBS), followed by disassembly to examine thrombus growth.

However, these approaches are not without limitations. Visual assessment, along with systemic and blood parameter monitoring, lacks sensitivity, potentially leading to either excessive thrombus formation or no thrombus growth by the time of experiment termination. Additionally, flushing the device with PBS and disassembling can dislodge thrombi, making it difficult to accurately determine their original growth locations.

Furthermore, the absence of continuous thrombus monitoring represents a major limitation, as terminating experiments at a single time point fails to capture the dynamic nature of thrombus growth. These approaches yield only global results for the MCS, similar to the standard hemolysis test outlined in ASTM F1841‐19 [8].

However, spatially resolved thrombus formation and its dynamics are of great relevance for improving pumps. Sakota et al. [9] have developed a thrombus monitoring technique using hyperspectral imaging with near‐infrared light but this method takes 20 s for one measurement point and has a low resolution of 250 × 250 pixel. Therefore, we are working on an approach for thrombus monitoring with a higher resolution. This is done using a thrombogenic two‐phase blood analog fluid, designated as “ghost blood (GB)”, which is a combination of a thrombogenic plasma‐based blood analog fluid developed by Clauser et al. [10] and ghost cells (GCs) [11, 12, 13, 14, 15]. GCs are hemoglobin‐depleted erythrocytes, resulting in diminished light absorption, thereby increasing the optical penetration depth (OPD). This, in turn, enables optical flow measurement through techniques such as particle image velocimetry (PIV). The integration of GB and PIV facilitates monitoring of thrombus growth, offering new insights into MCS hemocompatibility.

Laser light used in typical PIV applications has a very limited OPD in blood because of the high absorbance and scattering in the visible spectrum [16, 17]. For blood with a hematocrit of 40%, the penetration depth is limited to just 100 μm [18]. Therefore, PIV is usually conducted with a translucent blood analog fluids, such as a water‐glycerol mixture with a Newtonian viscosity of 3.65 × 10^−6^ m^2^/s [19]. A modification of this fluid involves the addition of xanthan gum, which imitates the shear‐thinning effect of blood [19, 20, 21, 22, 23, 24, 25, 26]. However, a two‐phase blood analog fluid composed of GCs and PBS more closely resembles blood, as introduced by Jansen et al. [14] and Einav et al. [15]. This fluid enables an OPD of 12.6 mm for laser light [27] but neglects the coagulation behavior achieved by using plasma instead of PBS.

We hypothesize that a high‐resolution optical thrombus growth detection is possible using PIV with GB. Therefore, we validate PIV with GB by comparing it to existing results from the literature. We quantified the limits of OPD for PIV with GB, and finally tested thrombus growth detection in a centrifugal blood pump.

## 2 Methods

To validate and quantify PIV with GB, and to detect thrombi, we conducted three individual experiments with the FDA‐pump (Food and Drug Administration). This generic centrifugal blood pump was designed by the FDA and is used in the second multicenter round‐robin study to improve the accuracy and comparability of simulations. This pump has been extensively studied in simulations and validated in experiments. Experimental validation was carried out using PIV and hemolysis experiments published by Hariharan et al. [28] and Malinauskas et al. [29], making the FDA‐pump a suitable model MCS for our two‐phase blood analog fluid GB [28, 29, 30, 31, 32].

**FIGURE 1:**
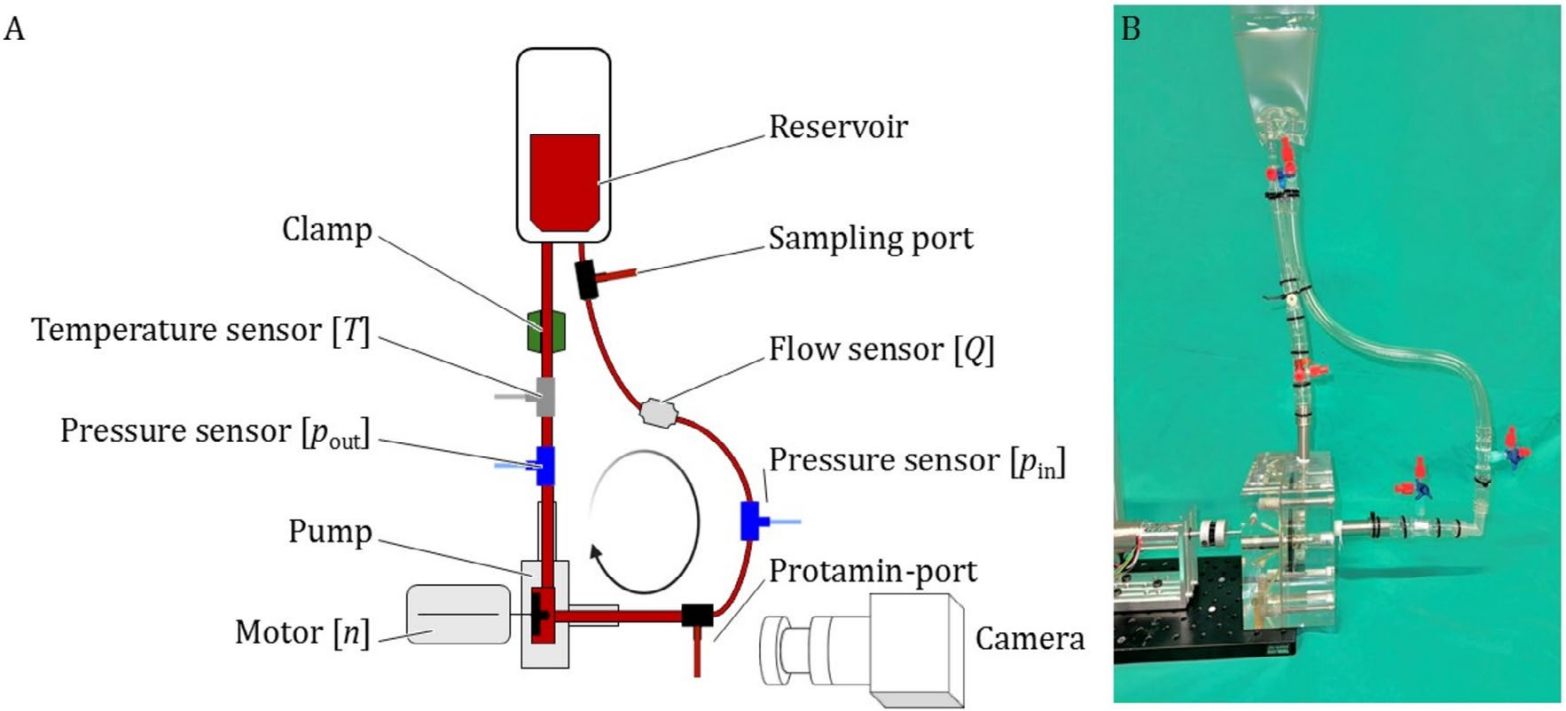
Experimental setup schematic showing the FDA‐pump and the connection of tubing to a blood bag. On the tubing sensors for temperature, flow and pressure are located. The camera has a frontal view on the pump and the laser is orthogonal to the camera and is coming from behind in this schematic [28, 29]. [Color figure can be viewed at wileyonlinelibrary.com]

The FDA‐pump is connected to a blood bag via tubing, and it is primed with 134.6 mL of GB containing 42% GCs and platelet‐poor plasma (Figure 1). The GCs are produced according to the process described by Schuermann et al. [33], and the platelet‐poor plasma is extracted from fresh porcine blood acquired in a slaughterhouse by centrifugation at 4000 g for 15 min (Rotina 420 R, Hettich GmbH & Co. KG, Germany). The porcine blood is anticoagulated with a sodium citrate solution (3.13% Eifelfango, Bad Neuenahr‐Ahrweiler, Germany) at a 1:9 ratio and supplemented with 0.016 g/L Gentamycin (10 mg/mL Sigma, Taufkirchen, Germany). Additionally, the fluid is seeded with fluorescent particles (10.5 μm diameter) at a density of 10 particles per 32 × 32 pixel frame.

The FDA‐pump is positioned in a PIV setup (Figure 1) consisting of a laser‐excited plane (Nd:YAG EverGreen2, Lumibird SA, Lannion, France) and one orthogonal camera (FlowSense EO, Dantec Dynamics A/S, Skovlunde, Denmark). The camera is equipped with a 550 nm long‐pass optical filter and an AF Micro Nikkor 105 mm 1:2.8 D lens (Nikon, Tokyo, Japan). The plane of the rotor blades and the bifurcation at the diffuser is illuminated by the laser, with the camera positioned orthogonally from the inlet side. This positioning corresponds to “CS #2” [29, 34]. Multiple series of 400 double‐frame images are taken by the camera once per pump rotation at the same rotor position using a timing box and a light barrier. For each RPM setting, the time between laser pulses is adjusted to ensure that the displacement of fluorescent particles at the rotor tip is approximately 8 pixels, corresponding to 25% of the initial interrogation window size of 32 × 32 pixel.

The acquired double‐frame images are processed to generate velocity fields in Dynamic Studio (Dantec Dynamics, Skovlunde, Denmark) using adaptive PIV with five iterations and progressively smaller interrogation window sizes down to 16 × 16 pixel and a step size of 8 pixels. Cross‐correlation was executed via Fast Fourier Transform with a gaussian window function (*k* = 0.75). Peak validation was applied using a minimum peak height of 0.8, a peak height ratio of 1.2, and a signal‐to‐noise ratio of 3.0. Invalid vectors were excluded for the vector statistics using a universal median test in a 5 × 5 neighborhood, with a normalization threshold of 0.1 and an acceptance limit of 2.0. Subsequent image evaluations are performed using MATLAB (MathWorks, Natick, USA), and graphs are plotted in RStudio (Posit PBC, Boston, USA). For statistical relevance, the velocity fields are calculated from a minimum of 1000 double‐frame images. The background noise of the images is filtered by subtracting the minimal brightness of all images from each image.

### 2.1 PIV With GB vs. Newtonian Fluid

In a GB validation experiment, PIV results are compared to the data from Malinauskas et al. [29]. This data was obtained using a Newtonian blood analog fluid composed of 50%–53% saturated aqueous sodium iodide solution, 16%–17% glycerin, and 31%–33% water. The FDA‐pump operates at 3500 RPM and a volume flow of 2.5 L/min, referred to as “operation point 2” [29]. In our validation experiment, the image area, designated as CS #2, is adjusted to focus on the rotor blade tips at the image center, as this area is the primary region of interest and could not be imaged in the same field of view as Malinauskas et al. due to limitations on the optical penetration depth. Camera frequency is set to 15 Hz, while time between pulses is 8.5 μs and the exposure time is 30 μs. The physical interrogation window size for the validation experiment is 0.14 mm × 0.14 mm.

### 2.2 Optical Penetration Depth

In a quantification of OPD experiment, the signal‐to‐noise ratio (SNR) of the seeding particles and the peak height in the cross‐correlation map are determined. These parameters indicate the accuracy of the velocity field calculations in PIV. The OPD can be split into two components: the distance from the laser to the seeding particles (OPD_Laser_) and the distance from the seeding particles to the camera (OPD_Camera_). While OPD_Laser_ is calculated post‐acquisition, OPD_Camera_ is set within the experiment by shifting the laser‐exited plane orthogonal to different depths and capturing series of double‐frame images at each depth. The FDA‐pump enables a maximum OPD_Camera_ of 7 mm.

The SNR of the seeding particles and the peak height in the cross‐correlation map are calculated for 32 × 32 pixel (0.3 mm × 0.3 mm) windows in 10 images per OPD_Camera_ and 5 windows in each image per OPD_Laser_. The windows are positioned side by side along the left image border to maximize penetration depth while minimizing optical interference of the diffuser, rotor blades, or pump housing angles. The pump operates at 400 RPM and a volume flow of 0.1 L/min for the OPD experiment. As OPD is independent of pump speed, the lowest stable operating condition was chosen to ensure consistent circulation while minimizing shear. The camera is set to 6.5 Hz, the time between pulses is 160 μs, and the exposure time is 30 μs.

### 2.3 Thrombus Growth

A thrombus growth experiment in the FDA pump is visualized by combining GCs with heparinized platelet‐rich plasma, replacing the previously used citrated platelet‐poor plasma. This facilitates thrombus growth upon the addition of protamine. The priming volume is changed to 130 mL of 40% GCs, platelet‐rich plasma, and 0.0015 wt % xanthan (Sigma‐Aldrich, Germany) to enhance the viscosity at low shear rates. The platelet‐rich plasma is extracted by centrifugation at 140 g for 20 min from heparinized porcine blood, which is treated with 3500 IU/L unfractionated heparin‐sodium (B. Braun Melsungen AG, Germany), 0.9 g/L glucose (50% Glucose Intravenous Infusion B. Braun, Germany), 0.016 g/L gentamycin (Merck, Germany), and 0.1% isotonic saline (B. Braun, Germany).

Thrombus growth is induced by adding 20 IU protamine every 5 min at a speed of 100 IU/min. Thrombus presence is detected by a significant increase in mean image brightness, determined via a *t*‐test comparing 25 pre‐and post‐growth images. The area of the thrombus is calculated to assess its growth rate. In the thrombus growth experiment, the clamp was removed to allow unrestricted flow through the system and increase the likelihood of thrombus formation within the pump housing rather than upstream. This adjustment resulted in a naturally elevated flow rate of approximately 0.4 L/min at 400 RPM. The camera frequency is set to 6.5 Hz, the time between pulses is 150 μs, and the exposure time is 3000 μs.

## 3 Results

### 3.1 PIV With GB vs. Newtonian Fluid

**FIGURE 2:**
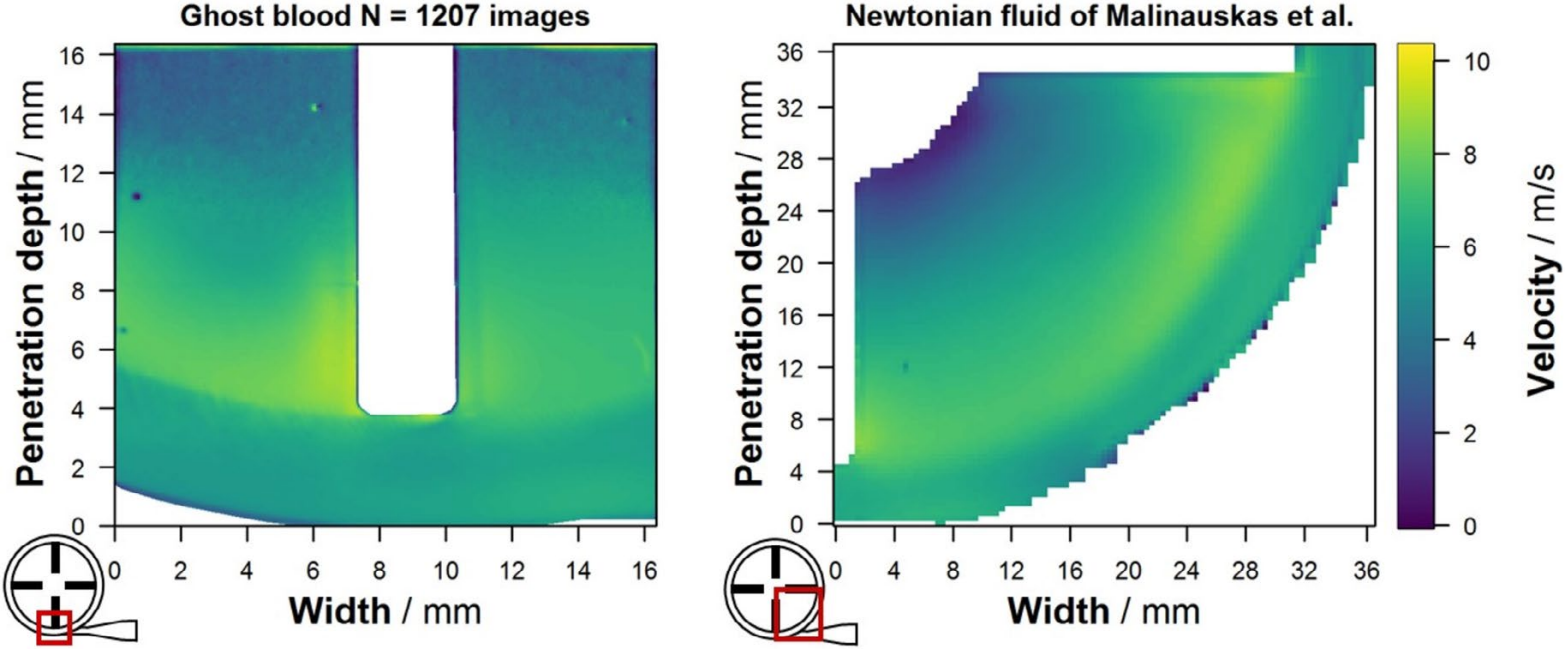
Velocity field measured by particle image velocimetry comparing ghost blood to a Newtonian blood analog fluid of Malinauskas et al. [29]. The maximum speed detected in ghost blood is 9.4 m/s and in the Newtonian fluid is 8.6 m/s. Velocity fields, standard deviation, and percentage of invalid vectors for different adaptive PIV settings are shown in the Supporting Information. [Color figure can be viewed at wileyonlinelibrary.com]

Figure 2 shows the validation experiment with the velocity fields calculated from PIV with GB, and the Newtonian fluid described by Malinauskas et al. [29] demonstrates a fairly similar velocity field. GB exhibits a maximum velocity of 9.4 m/s, while the Newtonian fluid exhibits a maximum velocity of 8.6 m/s. Furthermore, as OPD_Laser_ increases, a reduction in detected flow velocity and an increase in standard deviation are observed (not shown in graph). In comparison, the Newtonian fluid demonstrates a more uniform velocity transition between the rotor and the surrounding housing, whereas GB exhibits a more pronounced velocity gradient.

In this study, the densities of the tested fluids were as follows: porcine plasma, 1.0212 ± 0.0011 g/mL; ghost cell fluid (49.97% ± 3.19% HCT), 0.988 ± 0.021 g/mL; and whole blood (38.30% ± 2.65% HCT), 1.049 ± 0.001 g/mL.

### 3.2 Optical Penetration Depth

**FIGURE 3:**
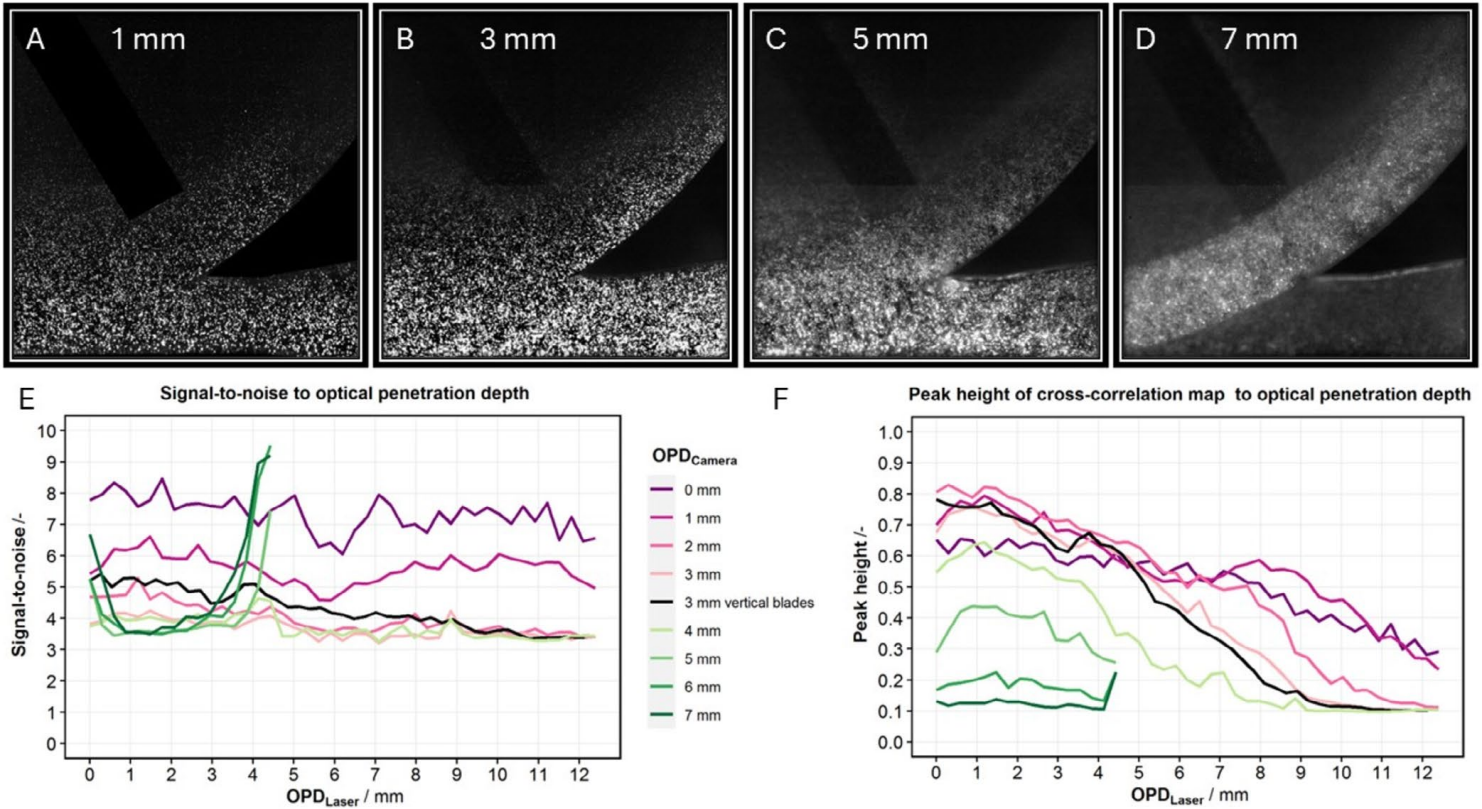
Signal‐to‐noise ratio and peak height of cross‐correlation map in particle image velocimetry depend on optical penetration depth through ghost blood from the laser to the camera. For the depth of 3 mm, the rotor blade is vertical as imaged in Figure 2. [Color figure can be viewed at wileyonlinelibrary.com]

For the OPD experiment, the signal‐to‐noise ratio (SNR) and peak height are calculated for an OPD_Camera_ up to 7 mm and for a OPD_Laser_ up to 12.4 mm. As OPD_Camera_ increases, seeding particles become increasingly blurred, leaving only the brightest particles distinguishable (Figure 3A–D). For an OPD_Camera_ larger than 5 mm, reflections on the rotor and pump housing result in high SNRs both for the low and high OPD_Laser_.

Focusing on the initial 4 mm of OPD_Camera_, the SNR (Figure 3E) decreases from 8.4 to 3.2, with increasing depth while the SNR for increasing OPD_Laser_ decreases slightly with slopes between −0.034/mm (*R* ^2^ = 0.079) and −0.11/mm (*R* ^2^ = 0.77). Similarly, the peak height of the correlation map (Figure 3F) decreases from 0.82 to 0.097 with increasing OPD_Laser_ and OPD_Camera_. The slopes are between −0.029/mm (*R* ^2^ = 0.90) and −0.065/mm (*R* ^2^ = 0.97).

### 3.3 Thrombus Growth

**FIGURE 4:**
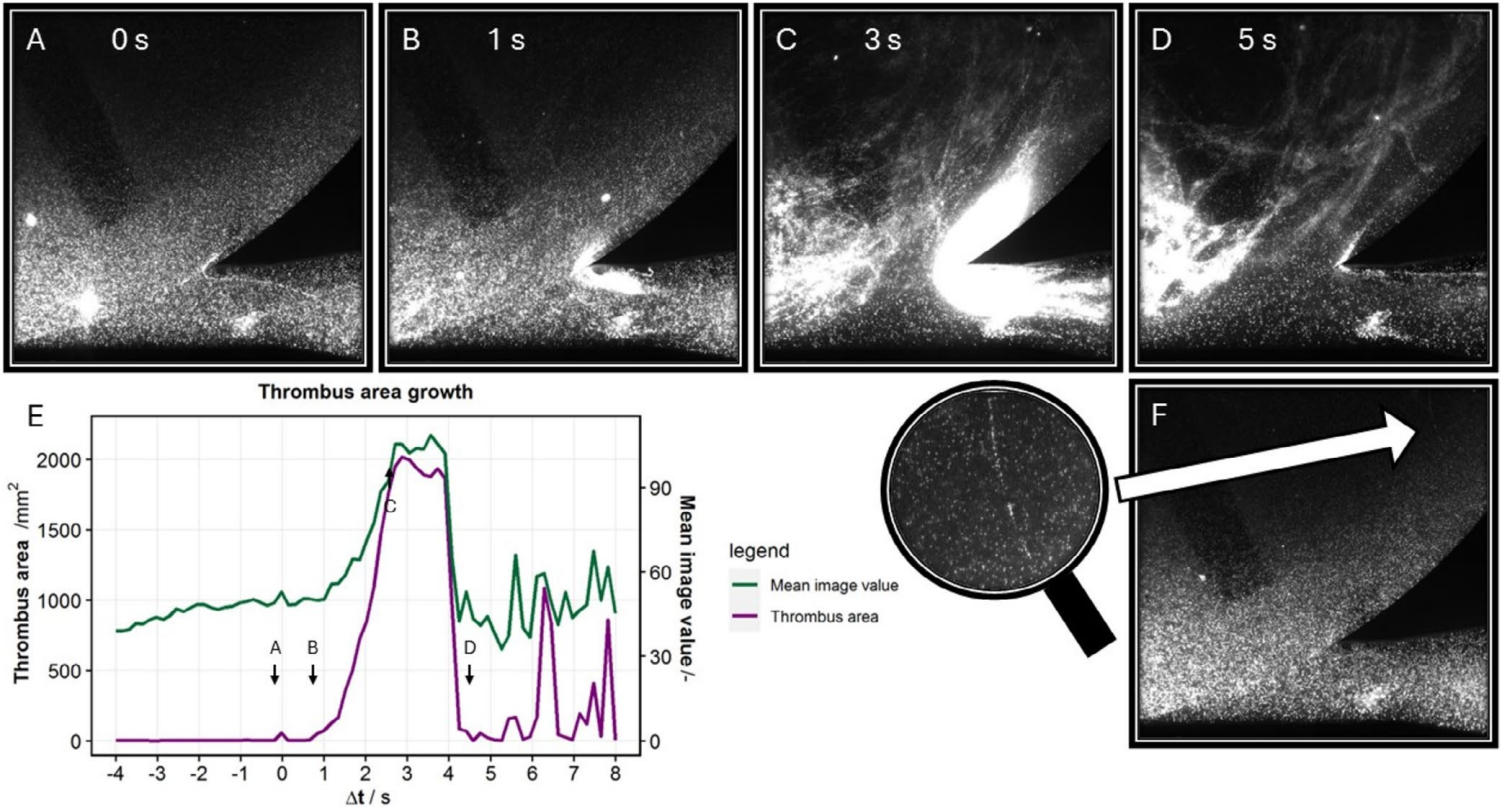
Thrombus area growth in a 3 s time span of a 28 min experiment. A‐D show the increasing thrombus growth over time; in D, the thrombus is pulled into the blades. E plots the calculated area of the thrombus and the mean image values. Before the thrombus event, there is already a single thread of accumulated seeding particles visible in F (*t* = −0.16 s). [Color figure can be viewed at wileyonlinelibrary.com]

Thrombus growth was first observed 28 min after the start of protamine addition, when a total of 120 IU protamine was already added. The thrombus accumulates seeding particles, exhibiting greater brightness than the surrounding fluid. Initially, a single tread becomes visible at the diffuser bifurcation (Figure 4A, *t* = 0 s). The output flow stops after 2 s (Figure 4C) and the maximum thrombus size (Figure 4A–C) is achieved within 3 s. A series of 25 images captures thrombus growth before the thrombus is dislodged and spun within the rotor (Figure 4D). The mean brightness (Figure 4E) increases significantly (*p* < 0.001) from the onset of thrombus growth compared to the preceding period. The area of increasing brightness, plotted in Figure 4E, illustrates the progression of thrombus growth over time (Video 1).

**VIDEO 1:** Frames 1–23 depict unobstructed flow of PIV particles within the FDA‐pump. Beginning at frame 24, thrombus formation is initiated at the bifurcation of the diffusor. Following frame 48, the thrombus detaches from the bifurcation and is carried into circulation within the pump. Video content can be viewed at https://onlinelibrary.wiley.com/doi/10.1111/aor.15042

In addition to the thrombus of maximum size, a secondary thrombus forms in the diffuser observed from the experiment's onset. The seeding particles within the secondary thrombus region exhibit blurring, and some particles persist throughout the experiment (Figure 4A–D). The secondary thrombus in the diffuser also shows an increase in brightness from Figure 4A–D.

A closer look at pre‐thrombus growth images (*t* = −0.16 s) reveals a trail of accumulated seeding particles within the rotor blade region (Figure 4F).

**FIGURE 5:**
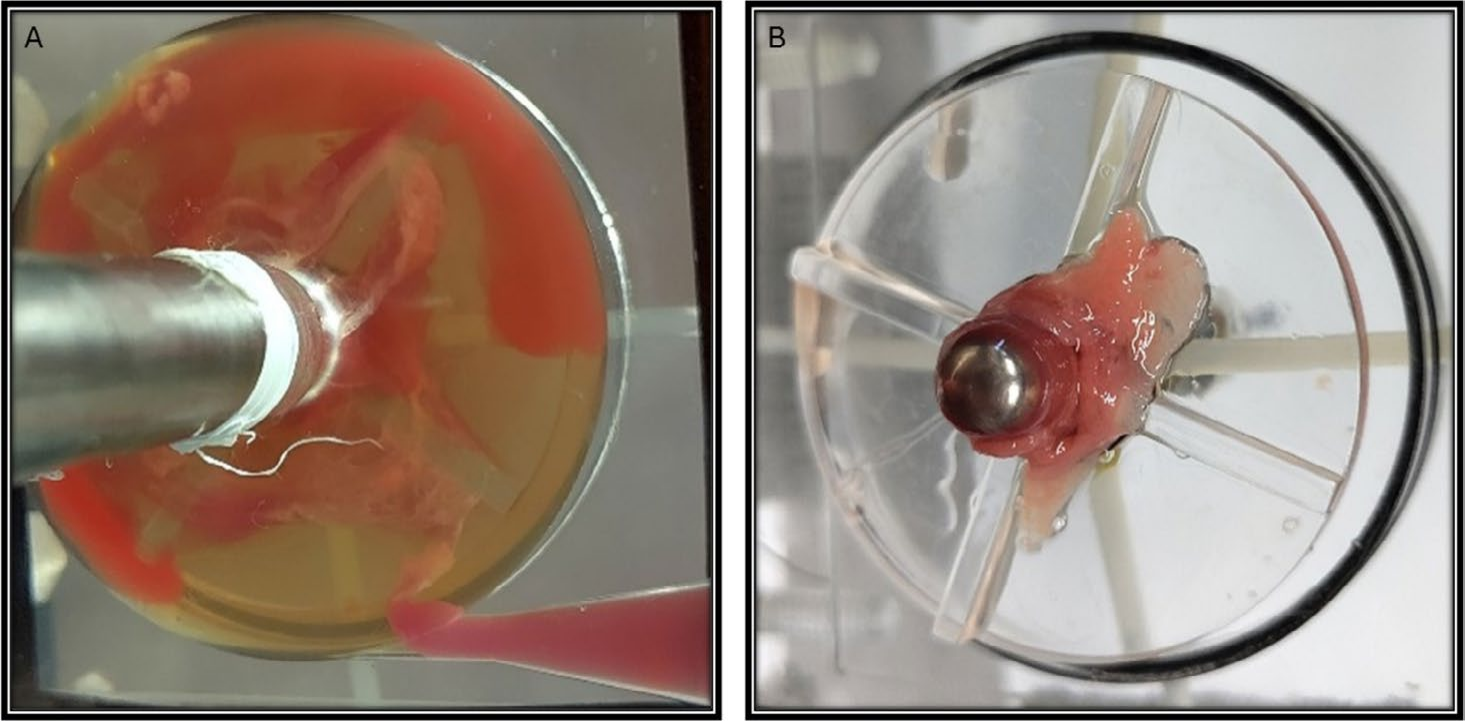
Resulting thrombus growth after experiment termination shows plasma, a white and a two red thrombus (A). Subsequently, to flushing, and disassembly, a color‐mixed thrombus adheres to the rotor center (B). [Color figure can be viewed at wileyonlinelibrary.com]

In this thrombus growth experiment, a white thrombus in plasma and two red thrombi are visible in the FDA‐pump (Figure 5). After flushing and disassembly, a mixed color thrombus was found adhered to the center of the rotor. The white thrombus was situated around the rotor with threads extending to the diffuser bifurcation, while the red thrombi were in the diffuser and in the housing surrounding the rotor.

## 4 Discussion

The overall velocity field measured in the FDA‐pump (Figure 2) in the validation experiment is consistent with the existing literature, thereby validating our PIV with GB. This includes the location of the maximum velocity just after the blade tip, the distinct velocity difference between the rotor area and the surrounding housing, and the gradual velocity decrease towards the pump center.

However, closer examination reveals notable differences. The maximum velocity for the Newtonian fluid was 8.6 m/s [29], whereas GB exhibited a maximum velocity of 9.4 m/s, similar to the calculated rotor tip speed of 9.5 m/s. While the Newtonian fluid's linear viscosity results in a more uniformly distributed velocity field, the shear thinning properties of GB, induced by the GCs, facilitate localized velocity increase. This same phenomenon explains the more pronounced velocity difference between the rotor area and the surrounding housing observed in GB. We suggest that GB more accurately mimics blood in this region with large velocity gradients compared to Newtonian fluids.

On the contrary, as OPD_Laser_ extends beyond 11 mm towards the rotor center, the velocity decreases, and unlike the smooth transition observed in the Newtonian fluid [29], there are higher variances between individual velocity vectors. This indicates a limitation in obtaining a feasible velocity field using PIV with GB, necessitating quantification which is done in the OPD experiment. At an OPD_Laser_ of 11 mm, the peak height of the correlation map decreased to a minimum value of 0.1. Therefore, we defined a peak height larger than 0.1 as a criterion for feasible velocity field measurements. Thus, OPD_Camera_ of 3, 4, and 7 mm result in a maximum OPD_Laser_ of 11.2, 9.1, and 4.1 mm, respectively (Figure 3). This results in a formula for the maximum feasible OPD_Laser_ as a function of the OPD_Camera_.

$${\mathrm{OPD}}_{\text{Laser}}=16.3\;\mathrm{mm}-1.75\times {\mathrm{OPD}}_{\text{Camera}}\;\left(R^{2}=0.99\right)$$

This function results in OPD values comparable to the 12.4 mm measured by Sikurova et al. [27].

On the contrast to peak height, SNR decreases to a minimum of 3.2 with increasing OPD_Camera_; however, this decline did not establish a definitive threshold when compared to the feasibility of the velocity field.

### 4.1 Thrombus Growth

This thrombus growth experiment presents an optical detection using particle image velocimetry. This was achieved using a blood analog fluid, Ghost Blood, mixed from Ghost Cells and platelet‐rich plasma. The visibility of the thrombus growth exceeded our expectations, as we initially expected only changes in PIV velocity fields to serve as indicators of thrombus growth. Accumulation of seeding particles to this extent was not expected. However, this accumulation significantly increased the mean brightness of the images during thrombus growth (*p* < 0.05). This increase in brightness enables the calculation of the thrombus area.

Secondary thrombus growth in the diffuser was observed from the experiment's beginning, and we hypothesize that it was caused by contamination of the pump from previous experiments. Unlike the primary thrombus, the secondary thrombus was not detected through seeding particle accumulation but appeared as a blurred region in the PIV images. It began to grow rapidly, accumulating seeding particles only after the primary thrombus started growing.

Other bright spots, such as in the lower left corner of Figure 4A, represent seeding particle accumulations likely caused by insufficient mixing or thrombogenic activity. However, these accumulations were visible in multiple images prior to thrombus growth and may represent initially adhered seeding particles.

The thrombus around the blades (Figure 4), characterized by seeding particle accumulation, resembled a white thrombus, whereas the thrombus formed in the diffuser, accumulating GCs, appeared more like a red thrombus. The thrombi visible in Figure 4 are consistent with findings from other thrombus analyses [9, 35, 36]. However, our method offers the added advantage of monitoring thrombus growth over time.

### 4.2 Limitations

During data processing, image time stamps indicate that the actual speed of the blades was approximately 360 RPM instead of the targeted 400 RPM.

For PIV, a transparent model of the MCS with the same refractive index as the fluid is required. This limits the application of the presented method in regulatory approval processes in MCS. The refractive index is generally matched by combining model material and adjusting the fluid's refractive index through fluid ratios (e.g., water and glycerol), adding refractive index‐matching substances, and changing the experimental temperature. However, no combination was identified that could adjust the refractive index without exerting cytotoxic effects on the GCs.

The FDA‐pump is manufactured from transparent acrylic and has a refractive index of 1.48. We measured the refractive index of GCs at a laser wavelength of 532 nm as 1.33746, using a 7‐wavelength refractometer (Schmidt+Haensch, Berlin, Germany). Such a refractive index mismatch can distort velocity field calculations.

The lower density of the GC‐based fluid is attributed to the removal of intracellular hemoglobin during ghost cell preparation. Nonetheless, the GC fluid presents a more physiologically representative density compared to commonly used water‐glycerol mixtures, which range between 1.600 and 1.750 g/mL as reported by Malinauskas et al. Therefore, despite its reduced density, the GC fluid offers a superior analog for cardiovascular flow studies. Moreover, due to the high hematocrit and cellular content, we expect sufficient particle‐cell interactions to ensure accurate flow tracking using fluorescent PIV particles.

Thrombus growth occurred within 3 s, which is unphysiologically rapid and magnitudes shorter than the activated clotting time (ACT) [37]. This accelerated thrombus formation may result from differences in the GC, pre‐activation of platelets due to handling prior to the experiment, or a hyper‐sensitivity of the GB to the added protamine. Future investigations should focus on assessing GB with respect to different coagulation cascade measurements, ensuring its comparability with whole blood. In addition, post‐growth histological analysis of the thrombus is recommended to gain deeper insight into its composition and the initiated coagulation cascade. While the FDA‐pump primarily investigates shear‐induced thrombus growth with GB, future studies should also monitor thrombus growth induced by flow stagnation.

## 5 Conclusion

Ghost cells as a two‐phase blood analog fluid exhibited a distinct velocity profile in PIV compared to the Newtonian fluid, indicating improved accuracy in the velocity field from optical flow measurements. In comparison to blood, the OPD of our two‐phase blood analog fluid is a magnitude greater, ranging over several millimeters rather than mere hundreds of micrometers.

For the first time, thrombus growth has been successfully monitored using particle image velocimetry with a two‐phase blood analog fluid, enabling the visualization of both thrombus location and its growth over time.

## Author Contributions

**Benjamin J. Schürmann:** concept, data collection, data analysis, statistics, interpretation, and drafting of the article. **Pia Creutz:** data collection. **Thomas Schmitz‐Rode** and **Ulrich Steinseifer:** critical revision of the article. **Johanna C. Clauser:** concept of the article, critical revision of the article.

## Ethics Statement

The work with porcine blood from a slaughterhouse does not necessitate ethics approval, but in‐house protocols for a controlled environment and disposal are followed.

## Consent

All authors gave their consent to publication.

## Conflicts of Interest

The authors declare no conflicts of interest.

## Supporting information


Figure S1.

## Data Availability

The authors have nothing to report.
