# Supplementary material for: Ghost Cells as a Two‐Phase Blood Analog Fluid—Optical Thrombus Growth Detection Using Particle Image Velocimetry
Source: Artif Organs. 2025 Jun 18;49(10):1532–9. doi: 10.1111/aor.15042 (PMC13020648; doi:10.1111/aor.15042)
Supplement: Supplementary file 2 — Figure S1. [file AOR-49--s001.docx]

# Supplementary materials

Video I: **Video of thrombus growth in the FDA Pump**. Image rate is 6.5 Hz at 400 RPM.


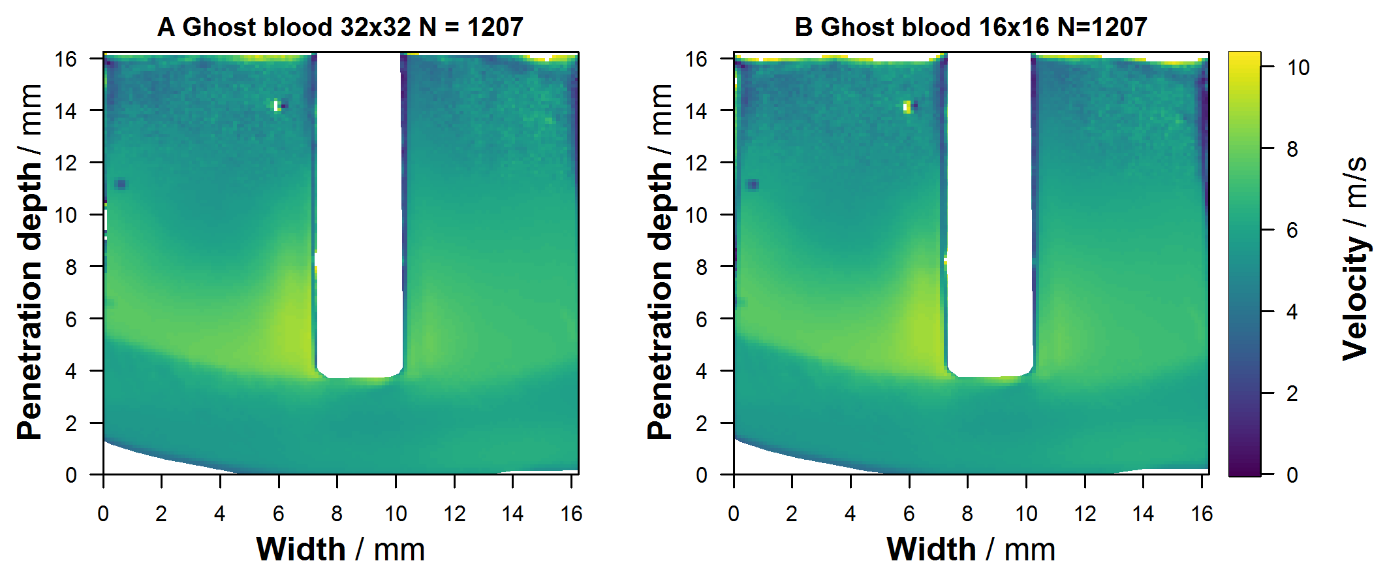

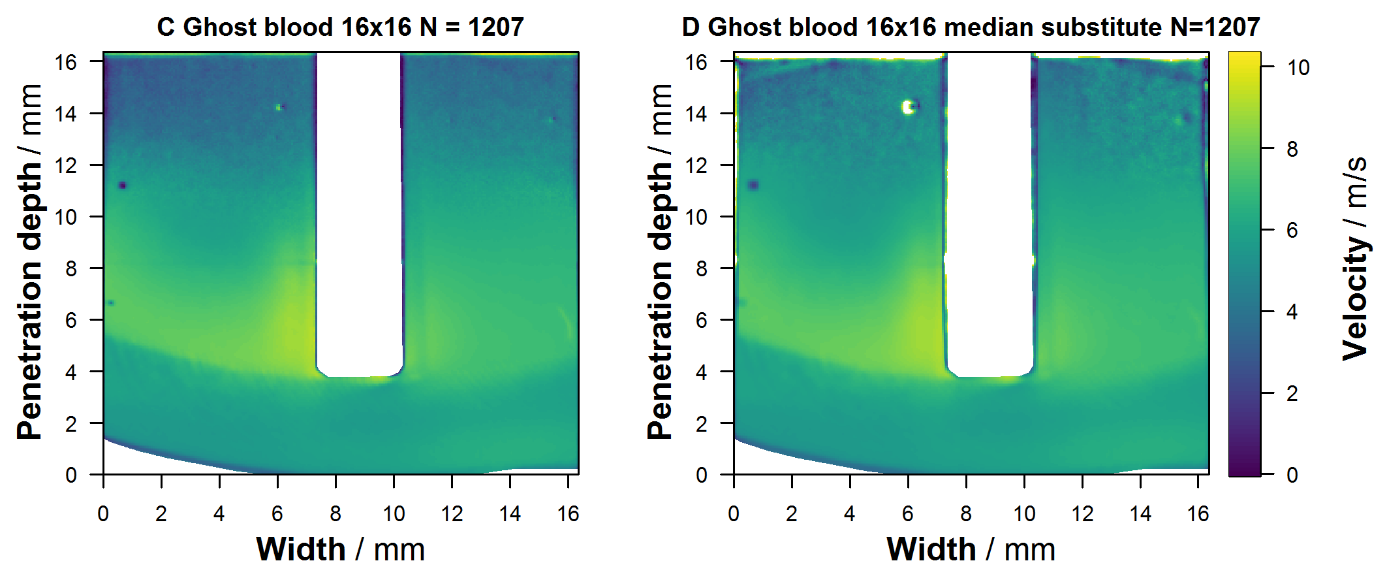


Figure I: **Velocity for different interrogation window size and step size**. A) interrogation window size 32x32 step size 16, B) interrogation window size 16x16 step size 16, C) interrogation window size 16x16 step size 8. A-C exclude invalid vectors from the vector statistics. D) interrogation window size 16x16 step size 8 with substituted invalid vectors. C) is the result presented in the manuscript.


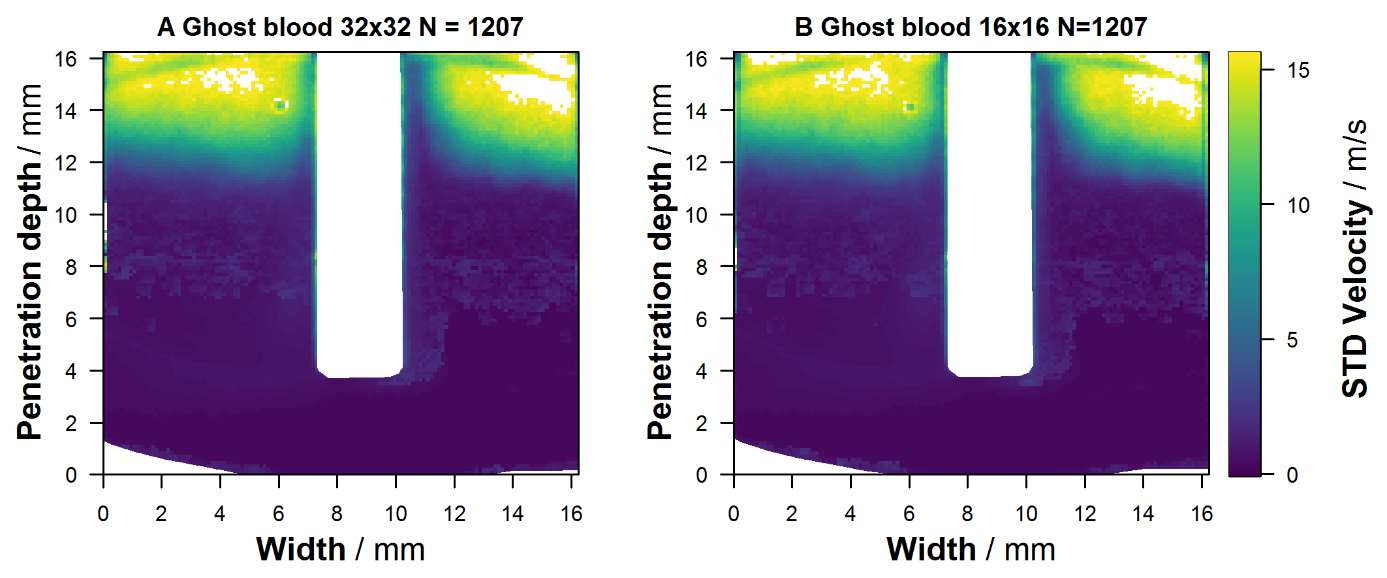

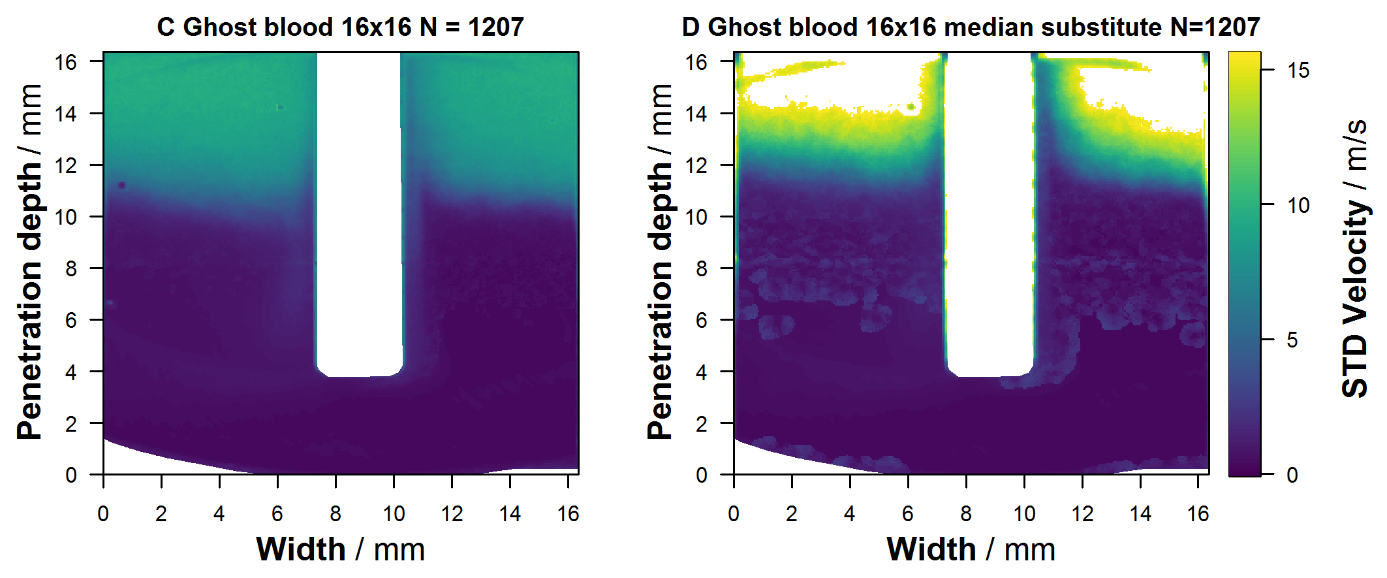


Figure II: **STD for different interrogation window size and step size**. A) interrogation window size 32x32 step size 16, B) interrogation window size 16x16 step size 16, C) interrogation window size 16x16 step size 8. A-C exclude invalid vectors from the vector statistics. D) interrogation window size 16x16 step size 8 with substituted invalid vectors. C) is the result presented in the manuscript. In the white areas the standard deviation is larger than 15 m/s.


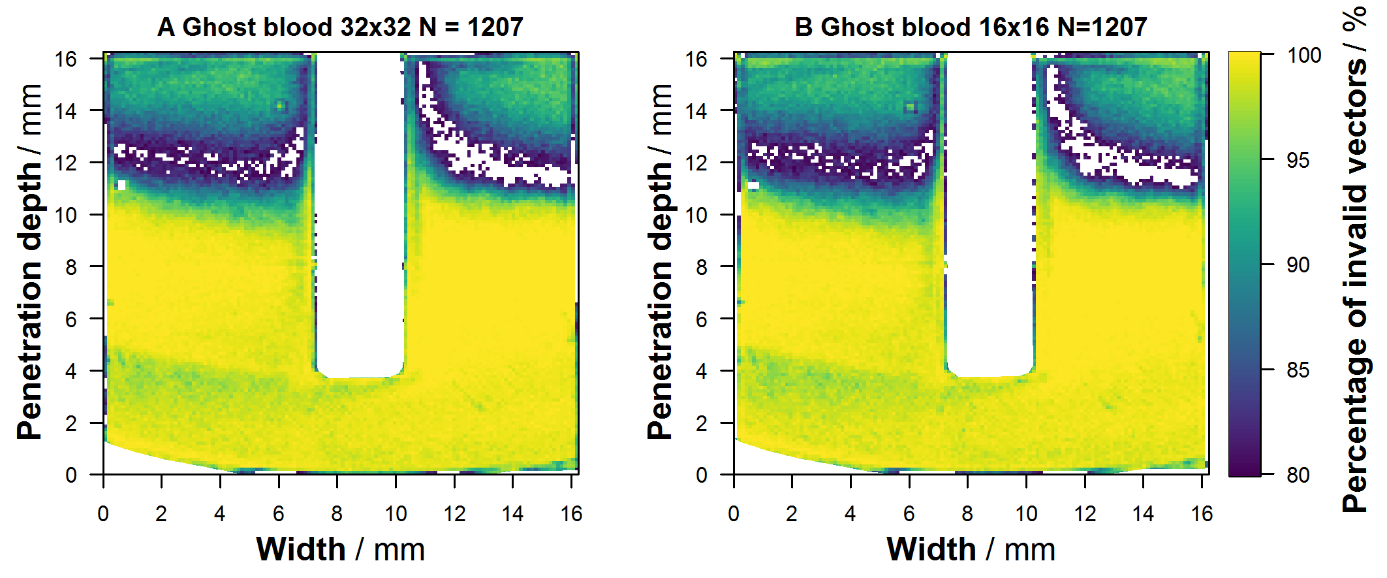

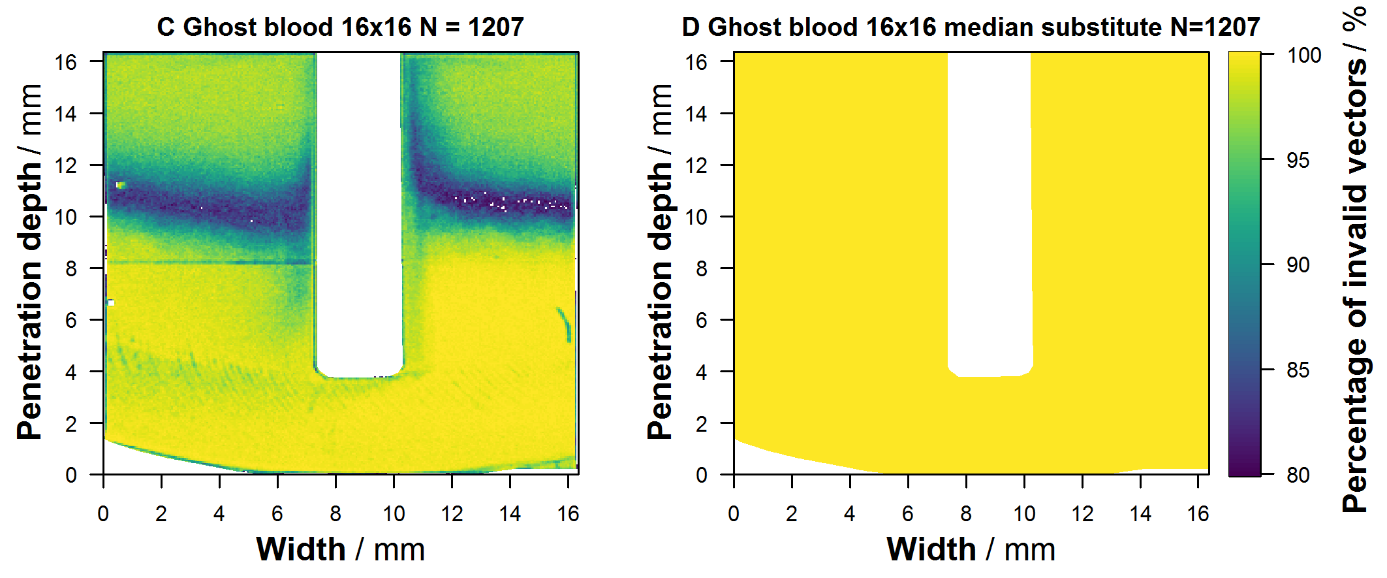


Figure III: **Percentage of invalid vectors for different interrogation window size and step size**. A) interrogation window size 32x32 step size 16, B) interrogation window size 16x16 step size 16, C) interrogation window size 16x16 step size 8. A-C exclude invalid vectors from the vector statistics. D) interrogation window size 16x16 step size 8 with substituted invalid vectors. C) is the result presented in the manuscript. In the white areas more than 20 % of vectors are invalid. Due to high standard diviatoins at high penetration depth the percentage of invalid vectors decreases again with increasing penetration depth over 10 mm.
